# Supplementary material for: Disruption of Sema3A/Plexin‐A1 inhibitory signalling in oligodendrocytes as a therapeutic strategy to promote remyelination
Source: EMBO Mol Med. 2019 Sep 30;11(11):e10378. doi: 10.15252/emmm.201910378 (PMC6835579; doi:10.15252/emmm.201910378)
Supplement: Supplementary file 1 — Appendix [file EMMM-11-e10378-s001.pdf]

Appendix

Disruption of Sema3A/Plexin-A1 inhibitory signalling in oligodendrocytes as a therapeutic strategy to promote remyelination

Fabien Binamé[1±], Lucas D. Pham-Van[1±], Caroline Spenlé[1], Valérie Jolivel[1], Dafni Birmpili[1], Lionel A. Meyer[1], Laurent Jacob[1], Laurence Meyer[1], Ayikoé G. Mensah-Nyagan[1], Chrystelle Po[2], Michaël Van der Heyden[1], Guy Roussel[1] and Dominique Bagnard[1*]

1- INSERM U1119 Biopathology of myelin, Neuroprotection, Therapeutic Strategy, Strasbourg University, Labex Medalis, Fédération de Médecine Translationnelle de Strasbourg.
2- Institut de Physique Biologique, Faculté de Médecine, Strasbourg University.

± Equal contribution

Table of content Page

Appendix Fig. S1: Expression of *Plexin-A1* and *SEMA3A* in multiple sclerosis patients versus healthy controls. 3

Appendix Fig. S2: Analysis of functional recovery with CatWalk assay. Data for hindlimbs 4

Appendix Fig. S3: MTP-PlexA1 reduces EAE relapse. 5

Appendix Table S1: Description of the human cohort of 8 healthy and 10 MS white matter samples. 6

Appendix Table S2: Validation of MTP-PlexA1 innocuity with blood analysis. 7

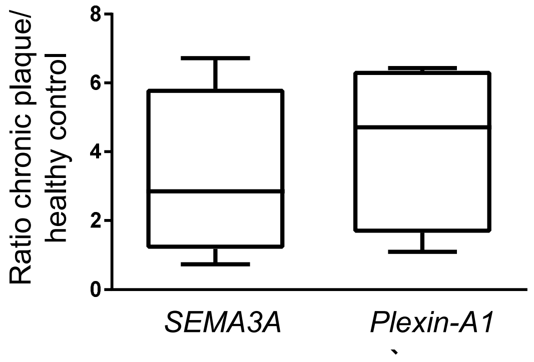

Appendix Fig. S1: Expression of Plexin-A1 and SEMA3A in multiple sclerosis patients versus healthy controls.
Expression level of *Plexin-A1* and *SEMA3A* in 4 chronic plaques over 2 healthy controls (Data extracted from the study of Han et al. 2012 using dataset GDS4218 analyzed at the GEO platform).

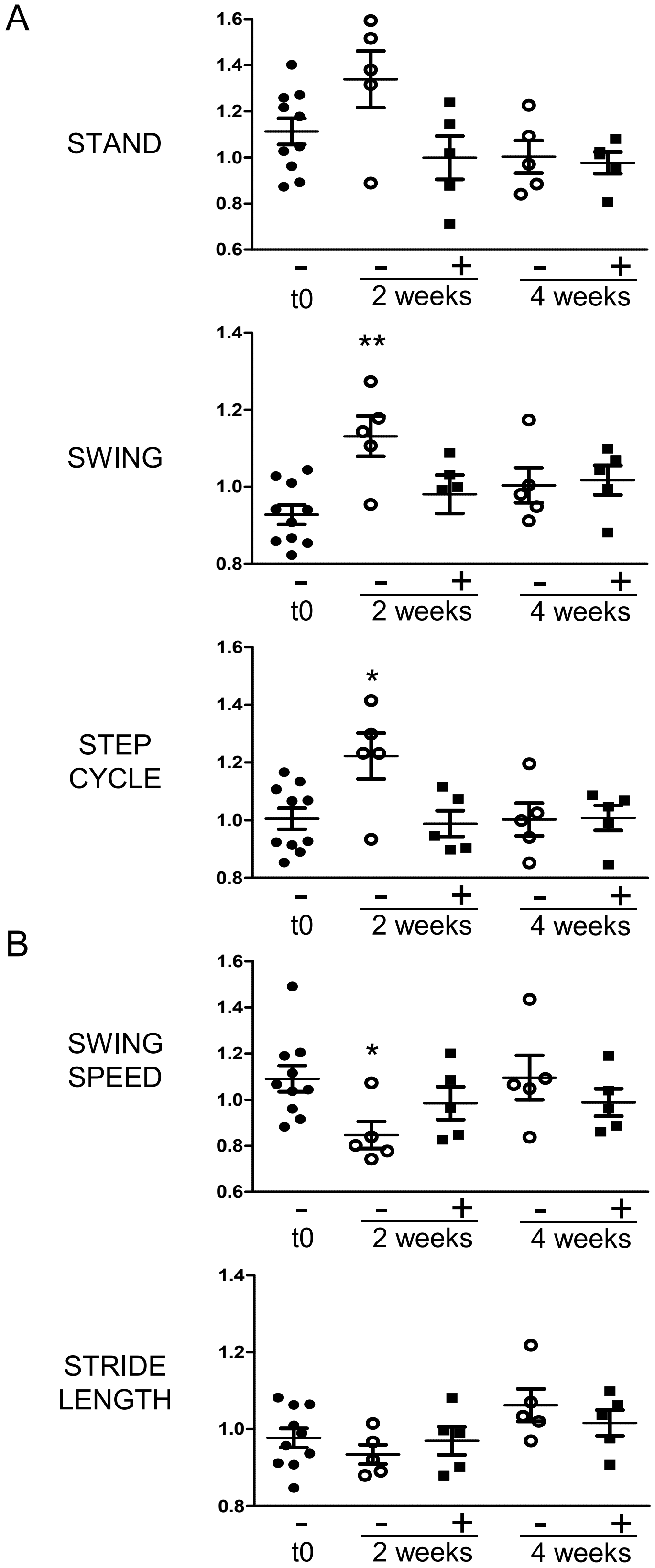

Appendix Fig. S2: Analysis of functional recovery with CatWalk assay. Data for hindlimbs

Gait analysis of mice fed 6 weeks with cuprizone diet then receiving a curative treatment with MTP-PlexA1 or vehicle concomitant with a normal diet. All parameters are expressed relatively to the end of cuprizone treatment (representing the last time without deficits) **(A)** Temporal parameters. (Mann-Whitney *p<0.05) **(B)** Kinetic and spatial parameters. Statistical significance is calculated toward values measured at the beginning of the experiment t0 (Mann-Whitney *p<0,05).

A

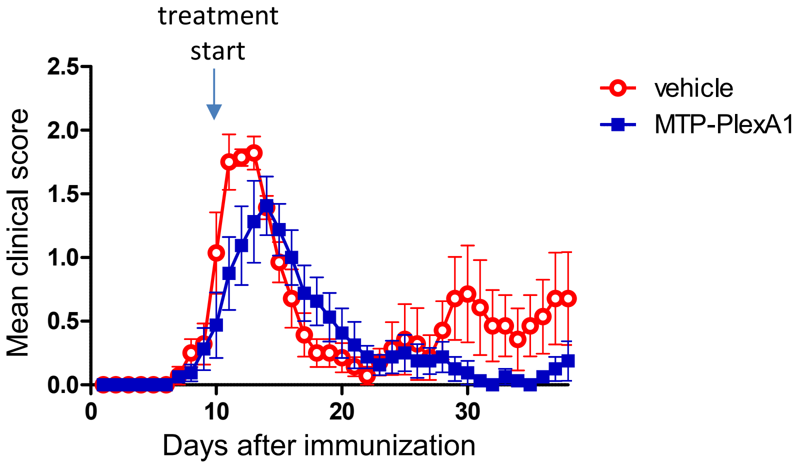

B

Treatment	Relapse percentage (score >= 2)	Chi-square test
vehicle	28.6%	*** P<0.0001
MTP-PlexA1 (1μg/kg)	0%	

Appendix Fig. S3: MTP-PlexA1 reduces EAE relapse.
SJL female mice were immunized with PLP (+ Pertussis toxin) and therapeutic treatment was administrated starting from day 10 (3 times per week) consisting of vehicle (n=7) alone or MTP-PlexA1 1μg/kg (n=8).
(A) Clinical disease profile. **(B)** Chi-square analysis of relapse percentage (score >=2).

	autopsy nbr	gender	age	pmd	diagnosis
1998-066	S98/153	f	75	04:50	Multiple sclerosis
1998-087	S98/183	f	55	07:35	Multiple sclerosis
2008-096	S08/295	f	88	07:55	Multiple sclerosis
2008-100	S08/302	f	77	10:00	Multiple sclerosis
2009-010	S09/034	f	40	09:00	Multiple sclerosis
2009-017	S09/054	f	68	08:25	Multiple sclerosis
2010-040	S10/110	f	57	08:40	Multiple sclerosis
2011-035	S11/035	f	50	07:35	Multiple sclerosis
2011-077	S11/077	f	66	09:35	Multiple sclerosis
2011-100	S11/100	f	71	07:05	Multiple sclerosis
1998-056	S98/123	f	83	05:15	Non-demented control
2002-024	S02/055	f	75	05:30	Non-demented control
2009-042	S09/134	f	84	06:55	Non-demented control
2011-049	S11/049	f	83	04:40	Non-demented control
2011-059	S11/059	f	84	06:05	Non-demented control
2012-005	S12/005	f	84	05:36	Non-demented control
2013-010	S13/010	f	89	06:35	Non-demented control
2014-053	S14/053	f	80	07:04	Non-demented control
2011-028	S11/028	f	81	04:25	Non-demented control
2010-034	S10/085	f	57	08:40	Multiple sclerosis

Appendix Table S1: Description of the human cohort of 8 healthy and 10 MS white matter samples. (pmd= post mortem delay)

Parameter	unit	vehicle	MTP-PlexA1	significance
White blood cells	10^3 cells/µL	2.23	2.07	n.s
red blood cells	10^6 cells/µL	9.46	9.6	n.s
hemoglobin	g/dL	13.25	13.45	n.s
hematocrit	%	52.6	52.7	n.s
mean corpuscular volume	fL	55.63	54.93	n.s
mean corpuscular hemoglobin	pg	13.98	14.08	n.s
mean corpuscular hemoglobin concentration	g/dL	25.13	25.6	n.s
granulocytes neutrophil	%	12.53	11.7	n.s
lymphocytes	%	83.93	84.2	n.s
monocytes	%	1.85	1.43	n.s
granulocytes eosinophil	%	1.15	2.38	n.s
large unstainded cells	%	0.38	0.08	n.s
granulocytes basophil	%	0.18	0.23	n.s
platelets	10^3 cells/µL	892.5	989	n.s
mean platelet volume	fL	4.6	4.58	n.s
Albumin	g/l	24	26	n.s
T.bilirubin	µmol/l	2.78	2.88	n.s
alanine amino transferase	U/l	21.8	16.4	n.s
alkaline phosphatase	U/l	107.2	124	n.s
creatinine	µmol/l	10.6	11.8	n.s

Appendix Table S2: Validation of MTP-PlexA1 innocuity with blood analysis. Haematology and clinical chemistry parameters of mice treated 4 weeks with MTP-PlexA1 or vehicle alone.
