# Supplementary material for: Disruption of Sema3A/Plexin‐A1 inhibitory signalling in oligodendrocytes as a therapeutic strategy to promote remyelination
Source: EMBO Mol Med. 2019 Sep 30;11(11):e10378. doi: 10.15252/emmm.201910378 (PMC6835579; doi:10.15252/emmm.201910378)
Supplement: Supplementary file 2 — Source Data for Figure 2 [file EMMM-11-e10378-s002.pdf]

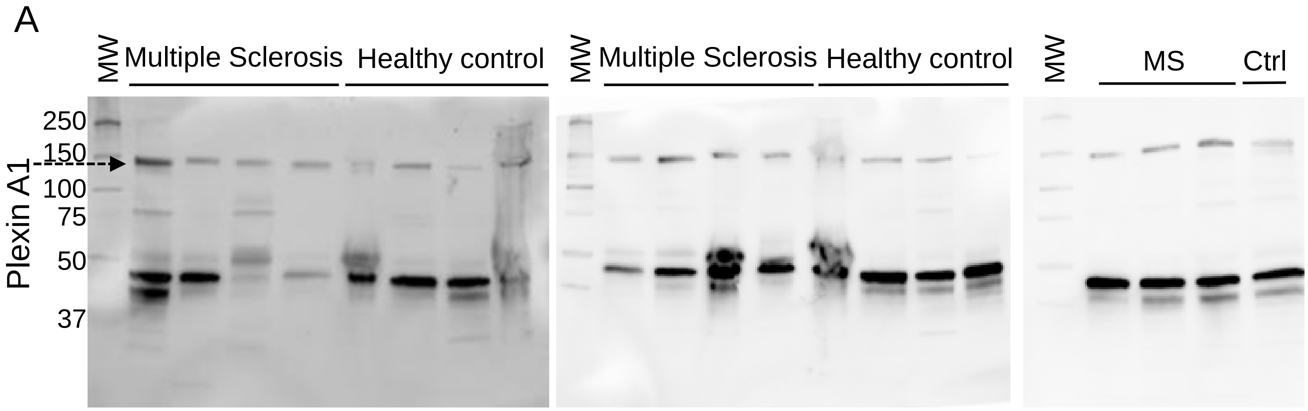

Source Data Figure 2: Expression of Plexin-A1 in multiple sclerosis patients versus healthy controls.

Full western blots showing brain samples lysates of multiple sclerosis patients and healthy controls revealed with anti-Plexin A1 antibody.(molecular weight = KDa)
